
JOURNAL INFORMATION
==============================
NLM Title Abbreviation: Biospektrum (Heidelb)
Journal ID: Biospektrum (Heidelb)
NLM Journal ID: 9615685

Biospektrum

ISSN: 0947-0867
EISSN: 1868-6249

ARTICLE INFORMATION
==============================
PMCID: PMC10230451
PMID: 37275943
DOI: 10.1007/s12268-023-1942-7
Article ID: 1942
Article version: 1
Subjects: Biotechnologie

Meilensteine in der Algenbiotechnologie

Griehl Carola 1
Schmid Andreas 2
Wilhelm Christian cwilhelm@rz.uni-leipzig.de http://www.lw.uni-leipzig.de/institut-fuer-biologie/abteilungen/algenbiotechnologie
3
1 grid.427932.9 0000 0001 0692 3664 Kompetenzzentrum Algenbiotechnologie, Hochschule Anhalt, Köthen, Deutschland
2 grid.7492.8 0000 0004 0492 3830 Department Solare Materialien, UFZ Leipzig-Halle, Leipzig, Deutschland
3 grid.9647.c 0000 0004 7669 9786 Institut für Biologie, Universität Leipzig, D-04318 Leipzig, Permoserstraße 15, Deutschland
Electronic publication date: 2023 May 31
Print publication date: 2023
Volume: 29
Issue: 3
First page: 306
Last page: 309
Copyright: © Die Autorinnen und Autoren 2023
License: Dieser Artikel wird unter der Creative Commons Namensnennung 4.0 International Lizenz veröffentlicht, welche die Nutzung, Vervielfältigung, Bearbeitung, Verbreitung und Wiedergabe in jeglichem Medium und Format erlaubt, sofern Sie den/die ursprünglichen Autor(en) und die Quelle ordnungsgemäß nennen, einen Link zur Creative Commons Lizenz beifügen und angeben, ob Änderungen vorgenommen wurden. Die in diesem Artikel enthaltenen Bilder und sonstiges Drittmaterial unterliegen ebenfalls der genannten Creative Commons Lizenz, sofern sich aus der Abbildungslegende nichts anderes ergibt. Sofern das betreffende Material nicht unter der genannten Creative Commons Lizenz steht und die betreffende Handlung nicht nach gesetzlichen Vorschriften erlaubt ist, ist für die oben aufgeführten Weiterverwendungen des Materials die Einwilligung des jeweiligen Rechteinhabers einzuholen. Weitere Details zur Lizenz entnehmen Sie bitte der Lizenzinformation auf http://creativecommons.org/licenses/by/4.0/deed.de.
License URL: https://creativecommons.org/licenses/by/4.0/

issue-copyright-statement: © Springer-Verlag GmbH Deutschland, ein Teil von Springer Nature 2023

==============================
Recent progress in algal biotechnology has identified new products based on their broad evolutionary origin. Novel metabolites were found for pharmacy, food industry, medicine e.g. tumor suppression and antibiotics. However, sustainable and economical algal production for crude oil replacement is limited by extremely low space time yields in photobioreactors. The consequences are a high energy burden for mass flow dependent processes and the need of space being in conflict with sustainable landscape management. New concepts using algae not as biomass producers but as living catalysts may open new options.

■ Die technische Nutzung von Algen als erneuerbare Rohstoffquelle rückt immer mehr in den Fokus von Wissenschaft und Wirtschaft, denn sie bauen schnell Biomasse aus $CO_2$, Sonnenlicht, Wasser und Nährstoffen auf, reinigen Abwässer und Abgase, binden Schwermetalle und liefern spezifische Wertstoffe für zahlreiche Anwendungen als Lebens- und Futtermittel, Kosmetik- und Pharmawirkstoff, Biokunststoff oder Nanomaterial.

Algen umfassen heute ca. 67.000 beschriebene Arten, die autotrophe Photosynthese betreiben und meist in Gewässern leben. So gehen ca. 45 Prozent der globalen Photosynthese auf Algen zurück. Ihre Bedeutung als $CO_2$-Senke ist mit der von Wäldern vergleichbar. Neben ihrer Leistung in der Wasserreinhaltung werden sie zunehmend industriell genutzt. Aufgrund ihrer großen phylogenetischen Diversität (**Abb. 1**) unterscheiden sich Stoffwechselwege und Produktspektren deutlich, mit enormen Konsequenzen für ihre technische Anwendung.

Die Biomasseerzeugung von Mikroalgen erfolgt in Suspensionskulturen entweder in offenen Becken oder in Photobioreaktoren (**Abb. 2**). Die Grundlagen hierfür wurden in den 1940er-Jahren in Deutschland gelegt. Die erste kommerzielle Produktion startete 1960 mit *Chlorella* sp. (Taiwan, Japan) in offenen Becken. Der erste industrielle Photobioreaktor mit 500 Kilometer Glasröhren ging 2000 (Klötze, Sachsen-Anhalt) in Betrieb. In Europa wurden in den letzten 15 Jahren ca. 370 Anlagen errichtet, darunter 33 in Deutschland, allerdings sind die erzeugten Mengen für eine energetische Nutzung marginal [1].

Bisher werden nur wenige Arten industriell für Lebensmittelzusätze, Futtermittel, Biodünger oder Kosmetika produziert. Zunehmend erobern hochwertige Inhaltsstoffe den Markt, wie ω-3-Fettsäuren, Carotinoide und Proteine. Seit kurzem werden Schuhe auf Mikroalgenbasis hergestellt.

Photobioreaktoren können auf wertlosen Flächen installiert werden. Ferner haben Algen gegenüber Pflanzen eine ca. 3-fach höhere photosynthetische Effizienz. Die damit verbundenen höheren Flächenerträge sind eine technologische Chance, allerdings sind Aussagen in der Literatur bis zu 100-fach höheren Erträgen schwer interpretierbar, da die Bedingungen für die Algenproduktion im Freiland kaum mit Erträgen klassischer Landwirtschaft vergleichbar sind. Daher fehlen belastbare vergleichbare experimentelle Daten. Wesentliche Hürden für große Algenfabriken sind hohe Produktionskosten und aufwändige Zulassungsverfahren.

Das Potenzial von Algen für neue Anwendungen liegt u. a. in ihrer Chemodiversität mit teils einzigartigen Verbindungen für die Pharma- und gesundheitsorientierte Lebensmittelindustrie. Algale Metaboliten entfalten im menschlichen Körper u. a. antivirale, antientzündliche, antioxidative und tumorsupressive Wirkungen oder können selektiv Enzyme blockieren, die mit spezifischen Krankheitsbildern assoziiert sind. Von etwa 10.000 Hoffnungsträgern, die in den Wirkstofflabors gescreent werden, landet im Durchschnitt nur eine Handvoll in der Apotheke. Bisher sind 10 Wirkstoffe auf Algenbasis auf dem Markt. Hierzu gehört Iota-Carrageenan (Carragelose), ein sulfatiertes Galactosepolymer aus Rotalgen, das als antivirales Nasenspray seit 2008 vermarktet

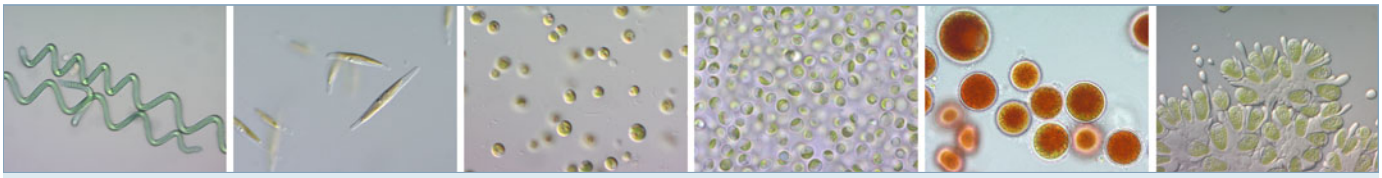

▲ **Abb. 1:** Mikroskopische Aufnahmen biotechnologisch genutzter Mikroalgen/Cyanobakterien. v. l. n. r. Cyanobakterium *Arthrospira platensis*, Kieselalge *Phaeodactylum tricornutum*, Eustigmatophyceae *Nannochloropsis* sp. und Grünalgen *Chlorella* sp., *Haematococcus pluvialis* und *Botryococcus braunii* (noch im Forschungsstadium. Fotos: CAB, Hochschule Anhalt.

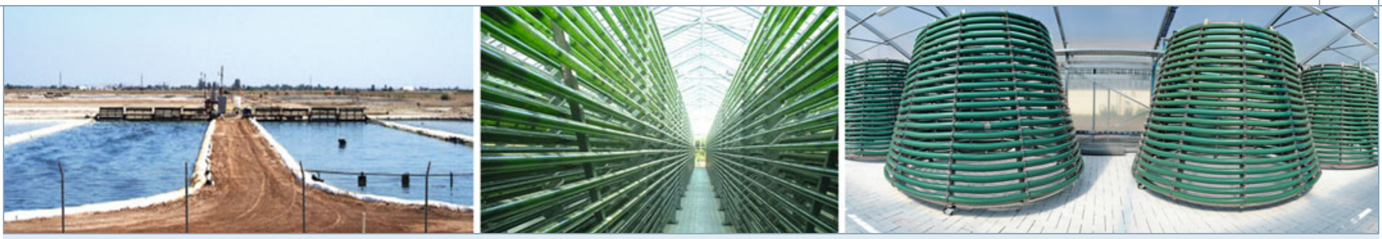

▲ **Abb. 2:** Open Ponds (Earthrise/USA), Glasröhren-Reaktoren (Klötze/BRD), GICON-Tannenbaumreaktoren mit Temperiersystem (Köthen/BRD), v. l. n. r.

wird und auch vor SARS-CoV-2-Viren schützt. Ein Oligomannuronat (GV-971) aus Braunalgen wurde 2019 in China zur Behandlung der Alzheimer-Krankheit zugelassen (Europa: Phase III). Der Wirkstoff hemmt Entzündungen und neuroinflammatorische Prozesse im Gehirn. Damit verbunden ist eine Reduktion der β-Amyloid-Ablagerungen und eine Verbesserung der kognitiven Fähigkeiten. Das aus Cyanobakterien isolierte Dolastatin-10 diente als Leitstruktur für die Entwicklung von Auristatinen. Diese werden in Form von Auristatin-Antikörper-Konjugaten erfolgreich zur Krebstherapie eingesetzt (**Tab. 1**).

Eine neue Wirkstoffklasse sind antivirale Lektine aus Rotalgen und Cyanobakterien. Sie binden spezifisch an Glykanketten der Virusproteine und inhibieren die Viren. Bekanntester Vertreter ist das Rotalgenlektin Griffithsin, das derzeit klinische Studien als Virostatikum gegen SARS-CoV-2, HIV, Hepatitis C und Herpes simplex durchläuft. Am ZNT in Köthen werden u. a. Glutaminylcyclase-inhibierende Sulfolipide als potente Therapeutika gegen Alzheimer [2] (DE102015011780.A1) und Paradontitis entwickelt.

Im Lebensmittelbereich sind zahlreiche Innovationen zu erwarten. Algen stellen ressourcenschonend ernährungsphysiologisch wertvolle Substanzen wie Proteine, Carotinoide, Vitamine und Mineralstoffe zur Verfügung, ganz im Trend gesunder Ernährung (Nestle-Zukunftsstudie). Vor dem Hintergrund einer wachsenden Weltbevölkerung stellt insbesondere die Proteinversorgung durch Algen wegen ihres Proteingehalts von z. T. mehr als 50 Prozent eine Chance dar. Damit können sie in Zukunft die Nische der Lifestyle-Produkte verlassen und Bestandteil der allgemeinen Ernährungspraxis werden. *Arthrospira* sp. (früher *Spirulina*) und *Chlorella* sp. werden schon jetzt verschiedenen Lebensmitteln beigefügt, wie Nudeln, Smoothies oder Süßigkeiten. Zur Steigerung der Nahrungsqualität und Stärkung der Immunabwehr können weitere algenspezifische Inhaltsstoffe zugesetzt werden, wie die ω-3-Fettsäuren EPA und DHA oder der Proteinfarbstoff Phycocyanin.

## Welche Probleme muss die Wissenschaft noch lösen, bevor Algen industriell nutzbar werden?

Algen können erhebliche Mengen an Speicherstoffen – wie Kohlenhydrate und Triacylglyceride – akkumulieren. Daher wird schon seit einigen Jahrzehnten die Idee vertreten, mittels Algen Biokraftstoffe oder andere Rohölersatzstoffe herzustellen.

Für Produkte, die einen Markt im Millionen Tonnenmaßstab bedienen sollen, spielen die Flächenproduktivität und der Flächenverbrauch eine entscheidende Rolle, da die Umnutzung von Flächen enorme ökologische und soziale Konsequenzen hat. So rechnete Flynn [3] vor, dass der Ersatz von nur zehn Prozent des Kraftstoffs für den europäischen Individualverkehr schon die Fläche von Belgien in Anspruch nehmen würde, wenn man die Algen in hochproduktiven Bioreaktoren produziert. Ein weiterer kritischer Punkt ist die in Photobioreaktoren geringe Volumenaktivität, die Raum-Zeit-Ausbeute (Produkt pro Reaktorvolumen und Zeit). Sie ist nach oben physikalisch durch eine maximal erreichbare Systemgrenze limitiert. Dies liegt am Energiegehalt des natürlichen Lichts. Zwar ist Licht in immensen Mengen verfügbar, aber aufgrund der Solarkonstante (1367 $W/m^2$) und von Abschwächungseffekten in der Erdatmosphäre nur sehr „verdünnt“. Je nach Breitengrad stehen zwischen 72,4 und 270,1 $W/m^2$ zur Verfügung. Der Faktor Licht begrenzt damit für phototrophe Prozesse direkt die Flächenausbeuten [4]. Biologisch ist die erreichbare Raum-Zeit-Ausbeute für Produkte lange vor dieser physikalischen Systemgrenze begrenzt – u. a. durch die Lichtempfindlichkeit der phototrophen Algen. Bulk-Chemikalien wären even-

**Tab. 1:** Antikörper-Wirkstoff-Konjugate (ADC) zur Therapie von Krebserkrankungen: zugelassene Präparate mit der Wirkstoffkomponente Monomethyl-Auristatin E und F (MMAE, MMAF), entwickelt auf Basis des Cyanobakterien-Oligopeptides Dolastatin-10. https://www.marinepharmacology.org/approved.

| Wirkstoff | Substanzklasse | Anwendungsgebiet/Indikation | Zielstruktur | Handelsname/Hersteller | Zulassung FDA (EU) |
|---|---|---|---|---|---|
| Brentuximab Vedotin | ADC (MMAE) | Hodgkin-Lymphom | CD30 & Mikrotubuli | Adcetris® Seagen/Takeda Pharma | 2011 (2020) |
| Polatuzumab Vedotin | ADC (MMAE) | Chronische lymphatische Leukämie, B-Zell-Lymphom | eEF1A2 | Polivy™ Genetech/Roche | 2019 (2020) |
| Enfortumab Vedotin | ADC (MMAE) | Urothelkarzinom | Nectin-4 | PADCEV™ Astellas Pharma/Seagen | 2019 (2022) |
| Belantamab Mafodotin | ADC (MMAF) | Multiples Myelom | BCMA | Blenrep™ GlaxoSmithKline | 2020 (2020) |
| Disitamab Vedotin | ADC (MMAE) | Blasenkarzinom, Magenkarzinom, Brustkrebs | HER2 | Aidixi™ China Remegen Biosciences | 2021 |
| Tisotumab Vedotin | ADC (MMAE) | Metastasierender Gebärmutterhalskrebs | Mikrotubuli | TIVDAK™ Seagen | 2021 |

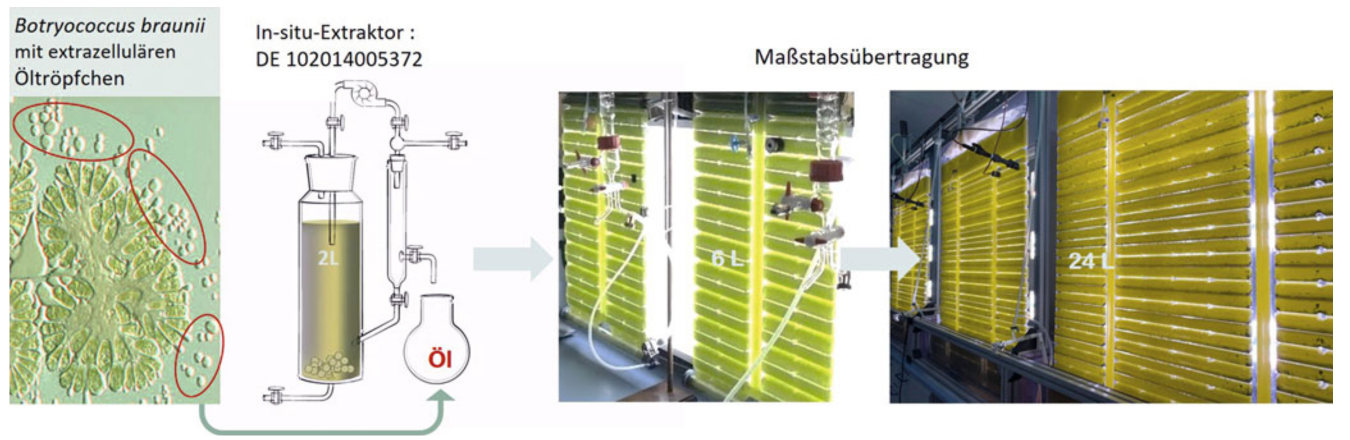

▲ **Abb. 3:** *Botryococcus braunii*, Prinzip Milking-Extraktor, Maßstabsübertragung in 6-L- und 24-L-FPA. v. l. n. r.

tuell über ein Numbering-up (viele dezentrale Produktionsanlagen) zugänglich, da auch die realisierbaren Bioreaktorgrößen anlagentechnisch begrenzt sind. Produktionsprozesse mit mixotroph oder heterotroph wachsenden Organismen nutzen hochkonzentrierte Energiequellen, wie z. B. Glucose oder organische Reststoffe, was höhere Raum-Zeit-Ausbeuten ermöglicht.

Ein Vergleich der Ethanolherstellung mittels transgenen Cyanobakterien mit heterotrophen Hefekulturen zeigt: Hefen können mit Glucose stündlich bis zu 33 kg EtOH pro $m^3$ bilden [5], während die besten bekannten Cyanobakterienstämme [6] nur 0,02 kg EtOH pro $m^3$ leisten. Damit sind Hefen ca. 1.650-mal leistungsfähiger als Cyanobakterien. Posten [7] gibt an, dass bei geschlossenen Algenphotobioreaktoren optimalerweise ca. 50 Liter Suspension pro $m^2$ Fläche anzunehmen sind. Dann braucht man 1,6-ha-Fläche, um mit den Cyanobakterien die gleiche Menge EtOH herzustellen, wie in einem 1-$m^3$-Hefefermenter auf Zuckerbasis. Rechnet man allerdings den landwirtschaftlichen Flächenbedarf für die Zuckerherstellung mit ein, ergibt sich im Vergleich eine ca. 8-fach höhere Flächenproduktivität der Cyanobakterien (Annahme: ca. 12 t Zucker pro ha, Algenproduktion an 200 Tagen bei einer durchschnittlichen Belichtungsdauer von 12 h). Diese Zahlen sind auf der Grundlage von publizierten biochemischen Effizienzen und nicht von technischen Anlagen berechnet. Ein analoges Ergebnis ergibt sich für die Nutzung von Cyanobakterien als Biokatalysatoren in Biotransformationen für die Herstellung von trans-L-4-Hydroxyprolin aus Prolin im Vergleich zu einem heterotrophen Prozess basierend auf rekombinanten *E. coli* [8, 9].

Aufgrund der geringen Volumenaktivität ergeben sich durch das Upscaling sehr große Volumina, die Managementprobleme für den Nährstoff- und Wasserbedarf zur Folge haben. Allein für die oben genannte Kraftstoffproduktion bräuchte man ca. 50 Prozent des Stickstoffs der gesamten europäischen Landwirtschaft. Die bei den großen Volumina entstehenden Stoffflüsse für die Reststoffverwertung der in der Algennährlösung zurückbleibenden Salze sind bislang noch nicht technisch umgesetzt, da sich die Algentechnologie noch auf der Pilotstufe befindet.

## Temperaturmanagement

Bei vollem Sonnenlicht im Sommer ergeben sich allerdings auch Probleme der Temperaturkontrolle. Da die Algen bei hoher Zelldichte die Solarenergie fast vollständig absorbieren und Photosynthese und Stoffwechsel nur einen Wirkungsgrad von wenigen Prozent haben, wird ein hoher Anteil der absorbierten Energie in Wärme umgewandelt und erhitzt die Suspension. Die verschiedenen technischen Kühlkonzepte setzen dafür entweder Wasser oder Energie ein, sind technisch aufwändig und stellen die Nachhaltigkeit in Frage. Ein erfolgversprechender Lösungsansatz ist der Einsatz eines geschlossenen Kultivierungssystems aus transparenten und flexiblen Kollektorschläuchen mit integriertem Kühlschlauch, der als Wärmetauscher fungiert [10] sowie die Wärmeabschirmung entweder durch IR-intransparente Filter oder PV-Folien, die zugleich einen Teil des Betriebsstroms liefern [11].

## $CO_2$-Neutralität

Aufgrund der mit industriellen Algenkulturen gegenüber Landpflanzen erzielbaren höheren Raum-Zeit-Ausbeuten sind die daraus zugänglichen Biomassen effektive temporäre $CO_2$-Senken, wenn zukünftig die $CO_2$-Nutzungseffizienz von aktuell 25–50 Prozent deutlich gesteigert werden kann. Bei der Bewertung der Kultivierungsprozesse müssen aber auch dabei anfallende $CO_2$-Emissionen aus der Gewinnung des Stroms für Pumpen, Kühlung, Ernte etc. berücksichtigt werden. Wenn für die Bereitstellung der Hilfsenergie keine erneuerbaren Energien eingesetzt werden, ist der Beitrag von Algenkulturen für die Klimaentlastung gering oder zuweilen sogar negativ [12].

## Lösungsansätze und deren technische Reife

Bei der Suche nach Lösungsmöglichkeiten werden verschiedene Alternativen verfolgt. Eine attraktive Möglichkeit ist die Nutzung der Algen als lebende Biokatalysatoren anstelle von Biomasse. So kann ein oxidiertes Produkt durch Photoreduktion in den Zellen zu hochwertigen Produkten umgewandelt und ausgeschieden werden. In diesem Fall nutzt man die Solarstrahlung für die Bereitstellung der chemischen Reduktionsreaktion [12].

Eine andere Möglichkeit besteht darin, algale Sekretionsprodukte zu verwerten. Intensiv geforscht wird an der Gewinnung von Kohlenwasserstoffölen (DE102014005372A1), Glykolsäure (DE102010040440A1) und Bioethanol (DE102014005372A1). Die Grünalge *Botryococcus braunii* bildet während des Wachstums erdölähnliche Kohlenwasserstoffe und sekretiert diese in eine extrazelluläre Matrix. Unter Einsparung der kostenintensiven Aufarbeitungsschritte Ernte, Entwässerung und

Zellaufschluss können diese Öle kontinuierlich aus der Algensuspension extrahiert werden (Milking) [14, 15].

Ein anderer Ansatz nutzt die Ausscheidung von Glykolsäure aus *Chlamydomonas* unter photorespiratorischen Bedingungen [16]. Durch Hemmung des Kohlenstoffanreicherungsmechanismus und des C2-Zyklus kann unter geeigneten Bedingungen erreicht werden, dass die Algen bei voller Photosyntheseleistung über Wochen kontinuierlich Glykolsäure im Medium bis zu Konzentrationen von 8 g/L anreichern [17]. Die Glykolsäure kann direkt weiter als Grundbaustein für die heterogene Katalyse oder als Monomer für Polyglykolat – ein wertvolles Polymer – genutzt werden. In diesem Fall ist die Nutzung des eingesetzten $CO_2$ nahezu vollständig und die Nährlösung muss nicht ersetzt werden, da die Nährsalze (N, P oder andere Elemente) nicht von den Zellen verbraucht werden. Zudem läuft der Prozess optimal bei 30 °C und verlangt daher weniger Kühlung [11].

Eine Alternative zu Suspensionskulturen sind Biofilme. Hier entfällt die Mischungsenergie und auch die Ernte ist deutlich vereinfacht. Allerdings sind bislang die Flächenproduktionsraten niedrig [18]. Im Biofilm kann ein steiler Lichtgradient die Produktion erniedrigen oder die Nährstoff- und $CO_2$-Versorgung limitieren. Hohe kurzzeitige Produktivitäten für Exkretionsprodukte lassen sich mit Mischkulturen in Kapillarsystemen erzielen [19].

Fortschritte sind vor allem dann zu erwarten, wenn es gelingt, hohe Lichteinträge in Suspensionsreaktoren einzubringen, z. B. über Glasfaser. In diesem Fall sind sowohl die Licht- als auch Nährstoffgradienten gut zu beherrschen und die Volumenaktivität könnte drastisch gesteigert werden. Dies sind aber auch aus ökonomischen Gründen Entwicklungen für das obere und mittlere Preissegment. Für großvolumige Produkte stehen experimentell testbare Systeme noch aus.

Ein vielversprechender Ansatz sind solare Bioraffineriekonzepte, die auf ein wirtschaftlich relevantes Produkt mit hoher Produktivität fokussieren und die Gewinnung mindestens eines weiteren Wertstoffs ermöglichen. In einer aktuellen Studie wird auf Basis experimenteller Daten mittels LCA-Simulation die Ko-Produktion von Proteinen und Biokraftstoffen für eine 500 Hektar große Mikroalgenproduktions- und Bioraffinerieanlage evaluiert [20].


Funding note

Open Access funding enabled and organized by Projekt DEAL.


Literatur

[1] Araújo R Vázquez Calderón F Sánchez López J Current status of the algae production industry in Europe: an emerging sector of the blue bioeconomy Front Marine Sci 2021 7 626389 10.3389/fmars.2020.626389
[2] Hielscher-Michael S Griehl C Buchholz M Natural products from microalgae with potential against Alzheimer’s disease: Sulfolipids are potent glutaminyl cyclase inhibitors Marine Drugs 2016 14 203 10.3390/md14110203 27827845 PMC5128746
[3] Flynn K (2017) Algal biofuel production is neither environmentally nor commercially sustainable. https://www-2018.swansea.ac.uk/press-office/news-archive/2017/algalbiofuelproductionisneitherenvironmentallynorcommercially-sustainable.php; Letzter Zugriff: 22.04.2023
[4] Franz A Lehr F Posten C Schaub G Modeling microalgae cultivation productivities in different geographic locations–estimation method for idealized photobioreactors Biotechnol J 2012 7 546 557 10.1002/biot.201000379 21751385
[5] Kachrimanidou V Vlysidis A Kopsahelis N Kookos IK Increasing the volumetric productivity of fermentative ethanol production using a fed-batch vacuferm process Biomass Conversion Biorefinery 2021 11 673 680 10.1007/s13399-020-00673-6
[6] Andrews F Faulkner M Toogood HS Scrutton NS Combinatorial use of environmental stresses and genetic engineering to increase ethanol titres in cyanobacteria Biotechnol Biofuels 2021 14 240 10.1186/s13068-021-02091-w 34920731 PMC8684110
[7] Posten C Design principles of photo -bioreactors for cultivation of microalgae Eng Life Sci 2009 9 165 177 10.1002/elsc.200900003
[8] Brandenburg F Klähn S Schmid A Krömer JO Produktion von Aminosäurederivaten in Cyanobakterien BIOspektrum 2022 28 341 343 10.1007/s12268-022-1756-z
[9] Brandenburg F Theodosiou E Bertelmann C Trans-4-hydroxy-L-proline production by the cyanobacterium Synechocystis sp PCC 6803. Metabol Eng Commun 2021 12 e00155 10.1016/j.mec.2020.e00155 PMC7815826 33511031
[10] Matthes S Matschke M Cotta F Reliable production of microalgae biomass using a novel microalgae platform J Appl Phycol 2015 27 1755 1762 10.1007/s10811-014-0502-4
[11] Nwoba EG Parlevliet DA Laird DW Pilot-scale self-cooling microalgal closed photobioreactor for biomass production and electricity generation Algal Res 2020 45 101731 10.1016/j.algal.2019.101731
[12] Daneshvar E Wicker RJ Show PL Bhatnagar A Biologically-mediated carbon capture and utilization by microalgae towards sustainable CO2 biofixation and biomass valorization–A review Chem Eng J 2022 427 130884 10.1016/j.cej.2021.130884
[13] Hoschek A (2019) Whole-cell redox biocatalysis driven by photosynthesis: an integrated bioprocess design for phototrophic biocatalysts. Chemical Biotechnology 31. Shaker, Aachen.
[14] Griehl C Kleinert C Griehl C Bieler S Design of a continuous milking bioreactor for non-destructive hydrocarbon extraction from Botryococcus braunii J Appl Phycol 2015 27 1833 1843 10.1007/s10811-014-0472-6
[15] Kleinert C Griehl C In situ extraction (milking) of the two promising Botryococcus braunii strains Showa and Bot22 under optimized extraction time J Appl Phycol 2022 34 269 283 10.1007/s10811-021-02633-7
[16] Günther A Jakob T Goss R Methane production from glycolate excreting algae as a new concept in the production of biofuels Bioresour Technol 2012 121 454 457 10.1016/j.biortech.2012.06.120 22850169
[17] Taubert A Jakob T Wilhelm C Glycolate from microalgae: an efficient carbon source for biotechnological applications Plant Biotechnol J 2019 17 1538 1546 10.1111/pbi.13078 30637910 PMC6662103
[18] Toepel J Karande R Bühler B Photosynthesis driven continuous hydrogen production by diazotrophic cyanobacteria in high cell density capillary photobiofilm reactors Bioresour Technol 2023 373 128703 10.1016/j.biortech.2023.128703 36746214
[19] Hoschek A Heuschkel I Schmid A Mixed-species biofilms for high-cell-density application of Synechocystis sp. PCC 6803 in capillary reactors for continuous cyclohexane oxidation to cyclohexanol Bioresour Technol 2019 282 171 178 10.1016/j.biortech.2019.02.093 30861446
[20] Karan H Roles J Ross IL Solar biorefinery concept for sustainable co-production of microalgae-based protein and renewable fuel J Cleaner Prod 2022 368 132981 10.1016/j.jclepro.2022.132981
